# Effects of a Proposed Hydraulic Project on the Hydrodynamics in the Poyang Lake Floodplain System, China

**DOI:** 10.3390/ijerph18158072

**Published:** 2021-07-30

**Authors:** Guizhang Zhao, Yunliang Li

**Affiliations:** 1 College of Geosciences and Engineering, North China University of Water Resources and Electric Power, 36 Beihuan Road, Zhengzhou 450045, China; guizhangzhao@163.com; 2 Nanjing Institute of Geography and Limnology, Chinese Academy of Sciences, Nanjing 210008, China; 3 State Key Laboratory of Hydrology-Water Resources and Hydraulic Engineering, Hohai University, Nanjing 210098, China

**Keywords:** floodplain system, 3D hydrodynamic modeling, outlet control, river–lake interaction

## Abstract

Knowledge of dam construction in floodplain systems and its hydrodynamic effects plays a critical role in managing various kinds of floodplains. This study uses 3D floodplain hydrodynamic modeling to explore the possible effects of a proposed hydraulic project in Poyang Lake (PLHP) on the hydrodynamics, exemplified by a large floodplain system. Simulations showed that the water levels across most lake regions presented more significant changes than in the floodplain areas during the study period. The increased water levels upstream from the PLHP (~1.0 m) were distinctly higher than that downstream (~0.1 m). The PLHP may decrease the magnitude of the water velocities in the main channels of the lake, whereas velocities may experience mostly minor changes in the floodplains, depending upon the altered flow dynamics and transport. On average, the water temperature may exhibit mostly minor changes (~<1.0 °C) for both the horizontal and vertical scales within the flood-pulse-influenced lake system. Additionally, the model results indicated that the outflow process caused by the PLHP may be altered from the natural discharge into the Yangtze River to frequent backflow events during the storage period, demonstrating the non-negligible effect of the PLHP on the water supply for the downstream Yangtze River in the future.

## 1. Introduction

Dams are constructed around the world to mitigate floods, support navigation, and store water for irrigation and hydropower generation [1,2]. Despite their various benefits, dams often disrupt the continuity and sediment transport in the natural water system of rivers and lakes, which in turn play a considerable role in affecting the geomorphological and hydrodynamic changes, as well as the associated ecosystems [3,4,5]. Dams have become one of the most concerning human interferences in natural systems as the number of dams and total storage capacity increase rapidly [6,7].

Dams may result in irreversible influences for both upstream and downstream ecosystems [8,9]. Dam operation can generate large effects on the water and sediment transport, water thermal regime, and associated water quality [10,11]. These changes are usually difficult to investigate and predict, with immediate and obvious properties [12,13]. Therefore, knowledge of dam construction and its regulating effects on hydrological and hydrodynamic behaviors has important implications for river and lake management and restoration.

In China, the Three-Georges Dam (TGD) has created many concerns about the benefits and impacts in the Yangtze River basin [2,8]. The TGD has provided benefits in terms of flood regulation, hydropower generation, and expanded river navigation capacity. However, it is also likely to lead to major changes to the downstream tributaries of the river and lake system, including Dongting Lake and Poyang Lake [8,14,15,16]. Poyang Lake is the largest freshwater lake in China, exhibiting a sensitive and dynamic response to rapid environment changes [17,18]. In recent decades, many previous studies discussed the consequence of the TGD’s effects. Reduced flows in the Yangtze River of the TGD during periods of reservoir filling enhance Poyang Lake’ outflows into the river (i.e., emptying effect) [15,16,19], directly leading to a reduced water level over the lake dry seasons [16]. Additionally, the lake has undergone frequent and severe drought in recent years, resulting in considerable impacts on the surrounding wetland ecosystem, drinking water supply, and irrigation around the lake [20].

Given the above background, the local government proposed a dam that linked the lake and the Yangtze River [10,11]. The dam with some designed floodgates is called the “Poyang Lake hydraulic project” (PLHP). The main purpose of PLHP is to alleviate the dryness of Poyang Lake during the low water level seasons, but it has received more debate among scientists in terms of the hydrological functioning and ecological responses (e.g., flow pattern, flushing time, thermal dynamic, and transport processes) [17,21]. In addition, the PLHP is more likely to produce unpredictable or irreversible impacts on the environment and ecosystem of the lake. In response to these cases, the regulation scheme of the PLHP has been adjusted many times by the local government; however, the controversy regarding the dam’s impacts is still ongoing [11,22].

**Figure 1:**
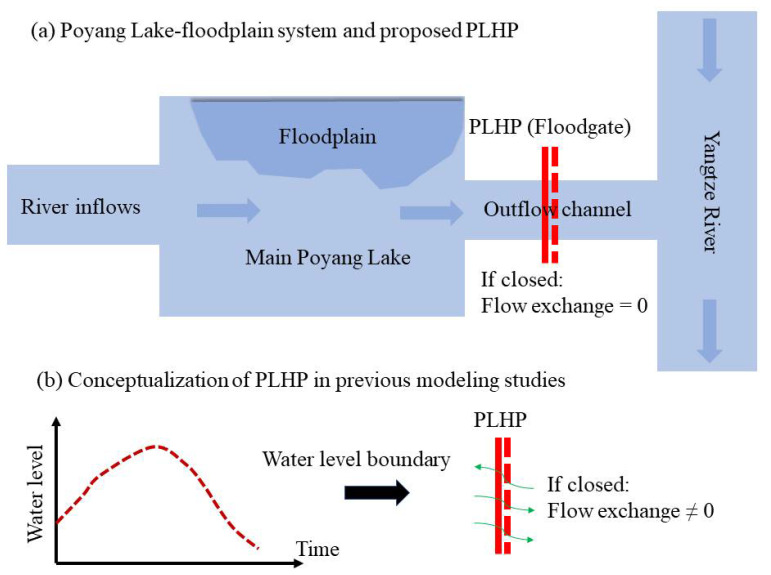
(**a**) Schematic diagram of the Poyang Lake-floodplain system and the proposed Poyang Lake hydraulic project (PLHP). (**b**) Conceptualization of the PLHP in previous modeling studies.

Hydrodynamic models can be used to assess various scenarios that are related to lake hydrodynamics and responses [23,24,25]. Several hydrodynamic models of Poyang Lake have been adopted and attempt to explore influences of the PLHP on the lake hydrodynamics. For example, Du et al. [26] used a 2D hydrodynamic model (finite volume method) to examine the PLHP’s effects on the lake’s hydrodynamic conditions and water quality responses. They found that the risk of eutrophication is likely to increase at some local regions of the lake. Hu et al. [27] used the depth-averaged EFDC (Environmental Fluid Dynamics Code) model to arrive at similar conclusions to those reported by Du et al. [26] regarding water quality trends generated by the PLHP. Wang et al. [10] and Lai et al. [11] employed the 2D EFDC model to predict the possible impacts of the PLHP on lake water level, flow velocity, and lake storage volume, indicating a disturbed hydrodynamic field after PLHP operation. In addition, Yang et al. [28] examined the influence of the PLHP on the hydrodynamics of the lake using a developed hydrodynamic model. They found that the PLHP may reduce the water velocity and, hence, increase the water age by up to around 9 days. It should be noted that these previous studies used water level changes as a boundary condition to represent the effect of PLHP within the models. A conceptual schematic diagram of the specified water level boundary for the PLHP is provided in Figure 1.

Previous modeling investigations regarding the effects of PLHP likely reflect the general trends of the lake condition, but the produced results may have some degree of uncertainty. That is, the adopted approach in these previous works [10,11,26,27,28] was incomplete or unreasonable because a water level boundary in hydrodynamic models allows for water flow exchange between the upstream and downstream of the dam, although the PLHP is closed. Recently, Tang et al. [29] pointed out the unrealistic description of the PLHP in hydrodynamic models. They employed a new method to conceptualize the PLHP by modifying the lake bottom elevation in a developed EFDC model. However, the method adopted in their work does not allow us to assess dynamic regulation scenarios that vary between seasons.

An improved understanding of the influence of the PLHP is essential for the Poyang Lake-floodplain management and planning. The objectives of this paper were to: (1) combine a 3D floodplain hydrodynamic model with a virtual hydraulic structure (i.e., floodgate) to conceptualize the PLHP; and (2) investigate the possible influences of the PLHP on lake hydrodynamics and thermal dynamics. The key innovation of the current work was the insight into a large Poyang Lake-floodplain system using a 3D hydrodynamic model and a new scenario to represent the PLHP.

## 2. Materials and Methods

### 2.1. Study Area

**Figure 2:**
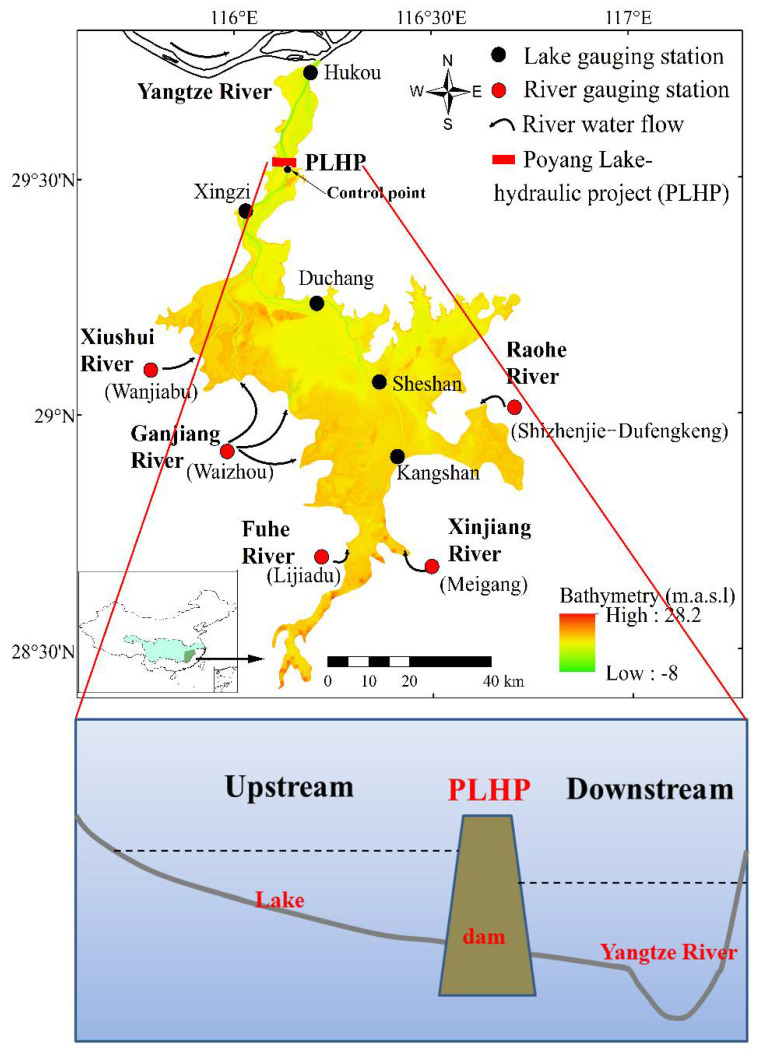
Topographic map (30 m × 30 m in year 2010) and major inflow rivers in the floodplain system of Poyang Lake. The lower panel shows schematic diagram of the PLHP in the river–lake system.

Poyang Lake is considered to be a large floodplain lake in the Yangtze River basin [14,16]. The lake is located at the south bank of the middle reaches of the Yangtze River (Figure 2). It is still freely connected to the upstream catchment rivers (e.g., Ganjiang, Fuhe, Xinjiang, Raohe, and Xiushui Rivers) and the downstream Yangtze River. The lake experiences a subtropical monsoon climate, with an average annual precipitation and evaporation of 1600 and 1000 mm/y, respectively [30]. Geographically speaking, the Poyang Lake floodplain system is characterized by complex morphology features, including main lake flow channels, extensive shallow floodplains, near lakeshore bays, and small islands [17]. The topographic elevations of the lake-floodplain system vary from −8 to 28.2 m (Figure 2). The water depth ranges from <6 m to 30 m during the flood inundation period [19]. The dramatic decrease in the lake water level during the dry period has coincided with the topographic changes. The volume of lake sand mining during 2000–2010 was approximately 6.5 times the volume of natural sediment that accumulated during 1955–2010 [19]. Within the lake-floodplains, the flow velocities range between 0.1 and 1.0 m/s [31] and the associated residence time vary from ~20–300 days [32]. The catchment rivers and the Yangtze River play a joint role in affecting the hydrological system, leading to different connectivity conditions between the lake and the adjacent floodplains (i.e., west–east direction) [33,34].

### 2.2. Overview of PLHP and Its Regulation Scheme

**Figure 3:**
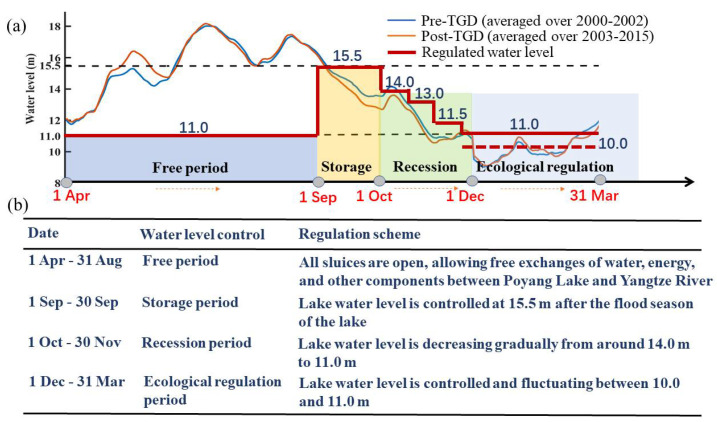
(**a**) Conceptual diagram showing the regulation scheme for the PLHP. (**b**) Corresponding lake water level changes of the PLHP under different periods, according to Lai et al. [22].

The PLHP (29°32′ N, 116°07′ E) was initially proposed by the Jiangxi Province Government in 2008 [10,11]. It is located in the northern outflow channel linking Poyang Lake and the Yangtze River (Figure 2). In order to alleviate the drought problems of the lake and to improve the lake ecological condition, a regulation scheme has been designed to control the water levels (e.g., Lai et al. [22]; Figure 3a,b). During the high lake water level seasons (from April to August), the PLHP remains open, thus allowing free exchange with the Yangtze River for water, energy, and biology. The PLHP only plays a role in adjusting a targeted water level from September to March (Figure 3a,b). In general, the regulation scheme can be divided into four major periods, including the free period, storage period, recession period, and ecological regulation period (Figure 3a).

### 2.3. 3D hydrodynamic Model

**Figure 4:**
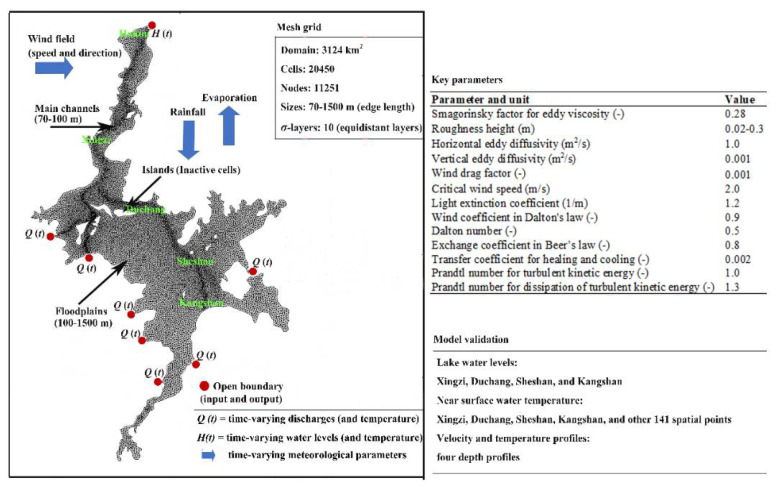
Computational domain, mesh grid, boundary conditions, key parameters, and model validation information for the MIKE 3 model of the Poyang Lake-floodplains, according to Li et al. [17].

In this study, the MIKE 3 model with an unstructured mesh is used to permit accurate representation of the complex characteristics of Poyang Lake [35]. The lake-simulated domain covers an area of about 3124 km^2^, with the element sizes varying from 70 m–1500 m (Figure 4). Regarding the vertical discretization, a total of 10 equidistant sigma layers were defined in the model [17]. The lake inflow boundaries were specified as time-varying river discharges, and the outflow boundary condition was specified as the water level observations (Figure 4). Air temperature, precipitation, wind speed, wind direction, relative humidity, solar radiation, cloud cover, and pan evaporation were specified as the atmospheric boundary conditions [17]. The initial values of water elevation were prescribed as the lake water surface using gauging observations (Figure 4), while the initial water velocities were set to zero throughout the model domain. To keep the model stability, the minimum time step was restricted to 0.1 s by trial and error. Additionally, the drying depth (0.005 m), flooding depth (0.05 m), and wetting depth (0.1 m) were used to follow the numerical option for wetting and drying in the MIKE 3 model. Key parameters used in the model are provided in detail by Li et al. [17], based on values from published studies on the lake and other modeling investigations (Figure 4). More details regarding the model construction can be found in Li et al. [17].

### 2.4. Model Validation and Performance

A MIKE 3 model validation of the Poyang Lake floodplain has been conducted using a variety of field observations of lake level and water temperature during 2015 (Figure 4), with the Nash-Sutcliffe efficiency coefficient (*E_ns_*) and determination coefficient (*R*^2^), both ranging from 0.96 to 0.99 [17]. In addition, a comparison between depth profile observations (i.e., velocity and temperature) and model simulations across the lake were conducted over the wet and dry seasons, demonstrating overall good agreement with reasonable accuracy [17]. These results indicated that the 3D floodplain hydrodynamic model is able to capture the dynamics in the water level, water velocity, and flow structure of Poyang Lake.

### 2.5. Conceptualization and Scenario Simulation

The 3D hydrodynamic modeling was selected to examine the impacts of the PLHP on lake hydrodynamics, based on two designed scenarios. The validated 3D model was used to represent the baseline condition, where the external factors followed an observed time series (i.e., natural condition without PLHP). Another scenario was adopted to simulate the effect of an unnatural-state lake (i.e., with PLHP). Since no water level station was constructed near the PLHP, we used control-point levels (Figure 2) designed for a floodgate [35] to conceptualize the regulation scheme of the PLHP (Figure 3) and, hence, to determine the open or close status (from water surface to lake bottom) of the PLHP. In the two scenarios, all external factors, except the condition along the PLHP, were the same. Therefore, the general idea was to identify the temporal and spatial differences between the hydrodynamic fields under the two scenarios to provide insight into the impacts of the PLHP throughout the lake-floodplain system.

The most notable reductions of the Poyang Lake water levels have occurred during particular times of the year (i.e., September–October; Figure 3a), mainly due to the combined effects of the TGD [8,16] and the lake sand mining [36,37]. Additionally, previous studies have pointed out that the PLHP is likely to be an effective way to balance the operation of TGD and the reduced water level of Poyang Lake (e.g., Zhang et al. [8,16]). Given these backgrounds, the present simulations only focus on the storage period from 1 September to 1 October (Figure 3), based on a normal year in 2015.

## 3. Results and Discussion

### 3.1. Impacts on Lake Water Level

**Figure 5:**
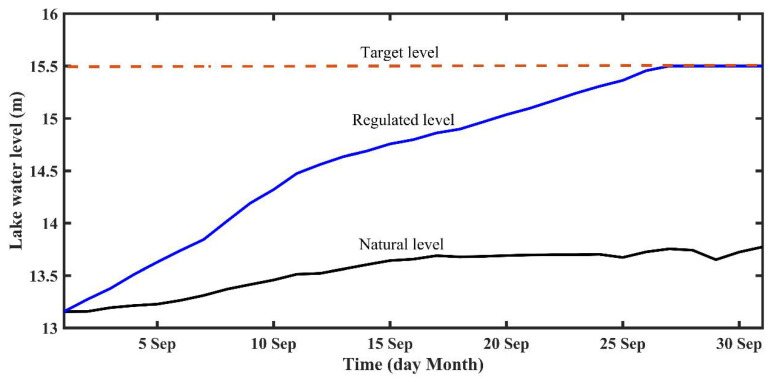
Time series changes in lake water levels at the control point of the PLHP for the natural and regulated conditions.

**Figure 6:**
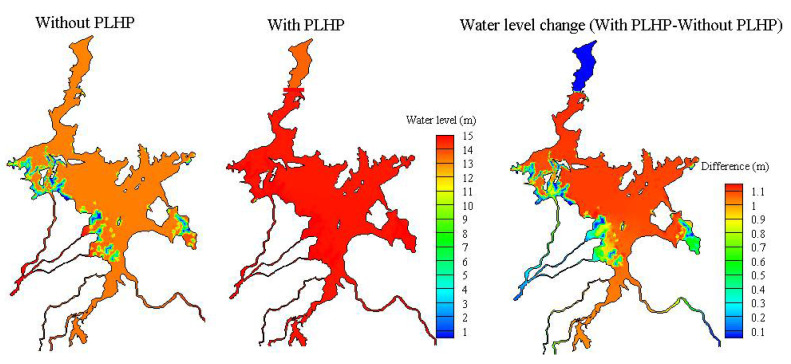
Influence of the PLHP on spatial water levels averaged over the storage period of the PLHP. Positive differences indicate that the water level in the scenario with PLHP is higher than the natural condition.

Figure 5 shows the temporal changes in lake water levels at the control point of the PLHP under the natural and regulated conditions. The simulation results revealed that the PLHP may increase the lake water levels and, hence, reach the targeted lake level (i.e., from 13.2 to 15.5 m) during the storage period. The effect of the PLHP on the spatial pattern of the water level changes in Poyang Lake is illustrated in Figure 6. The results indicated that the spatial variability of lake water levels was observed between the main lake and the floodplains (i.e., west–east direction) during the natural condition (without PLHP), while the spatial variability can be found between the upstream and downstream of the PLHP (i.e., south–north direction) for the regulated condition, indicating the PLHP played a target role. Generally, the effect of the PLHP on spatial water levels exhibited a distinct lake-wide pattern during the storage period. The simulations demonstrated that, on average, the water levels across the majority of the lake regions presented larger changes (i.e., up to 1.1 m) than in the floodplain areas (i.e., ~<0.5 m). We also found that the magnitude of increased water levels upstream of the PLHP (i.e., ~1.0 m) was distinctly higher than that downstream (i.e., ~0.1 m). This was an expected outcome given that the PLHP may substantially increase lake water levels in the upstream areas and promote frequent backflows from the Yangtze River downstream of the PLHP (see Section 3.3), hence leading to increasing water levels near the downstream outflow channels (Figure 6).

### 3.2. Impacts on the Hydrodynamic Field

**Figure 7:**
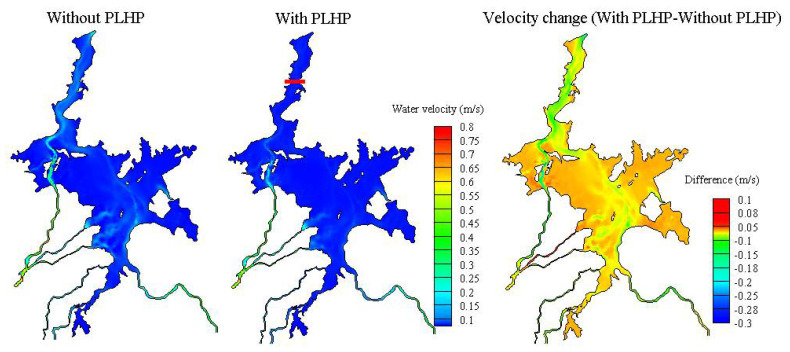
Influence of the PLHP on spatial water velocities averaged over the storage period of the PLHP. Positive differences indicate that the water velocity in the scenario with PLHP is higher than the natural condition.

Water velocity distributions and differences (averaged over the storage period) between the natural (without PLHP) and hypothetical conditions (with PLHP) are shown in Figure 7. Although the water velocity distributions under the two conditions appear to show a similar spatial pattern, the velocities in the flow channels during the natural condition were higher than those of the PLHP condition. The distributed effect of the PLHP on the velocities in the lake showed a complex response, based on the positive and negative changes of water flows to the PLHP. That is, the PLHP was likely to decrease the magnitude of the water velocities (i.e., <0.2 m/s) in the main lake flow channels, whereas velocities may increase (i.e., <0.1 m/s) in the surrounding floodplains. However, the floodplain areas may experience mostly minor velocity changes. Overall, the PLHP played a non-negligible role in affecting velocity responses in the downstream lake regions, as expected.

**Figure 8:**
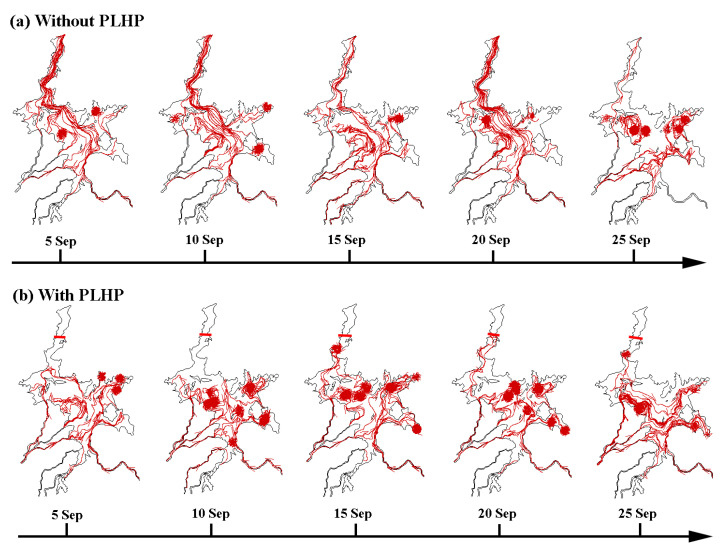
Influence of the PLHP on the spatial flow field based on velocity components and streamline (in dark red line) under the (**a**) natural condition and (**b**) hypothetical condition.

To clearly show the impacts of the PLHP on the hydrodynamics, Figure 8 depicts the hydrodynamic field and associated flow trajectories across the lake-floodplain system under the natural and PLHP conditions. During the natural condition, the water flows exhibited an overall northward movement from the catchment inflow rivers to the outlet, although eddies were observed at some local lake regions (Figure 8a). It is expected that the PLHP tends to block the normal water pathways and generate a reverse (southward) water flow across the lake-floodplains (Figure 8b). For example, a greater number of different eddies can be found in terms of eddy size and number, particularly for eddies occurring near the PLHP (e.g., 15 September and 25 September; Figure 8b). This reflected the important influence of the PLHP in modifying the flow dynamics and transport, resulting in substantial changes in flow direction and pathways within the lake. Additionally, the role of the PLHP on the Poyang Lake hydrodynamics was very similar to the effect of the backflow that disturbed the normal water flow dynamics [19].

### 3.3. Impacts on Lake Outflow and Backflow

**Figure 9:**
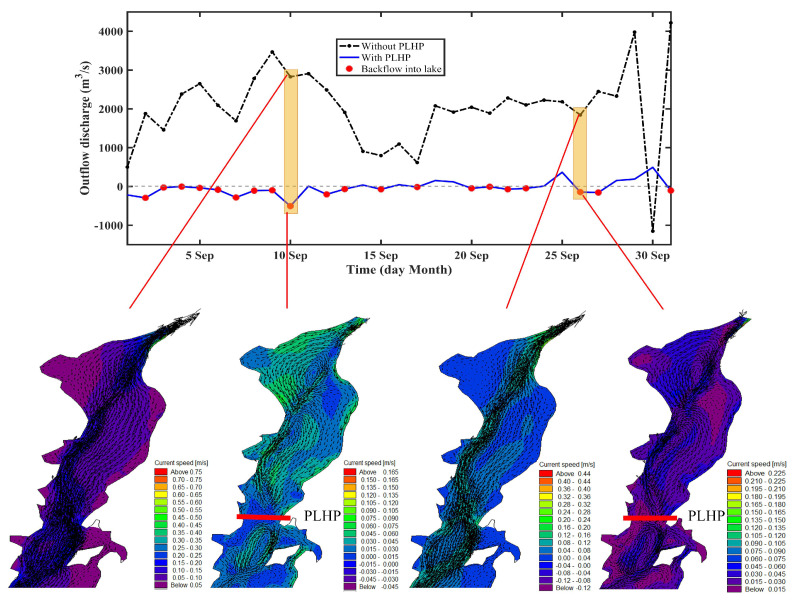
Influence of the PLHP on lake outflow discharges during the study period. The close up for the northern lake outflow channel (zoomed in) indicates the current vectors. Different velocity scales in the sub-panels aimed to distinguish velocity magnitudes for the two time slices.

The outflow of Poyang Lake is an important indicator for exploring the impacts of the PLHP on the lake–Yangtze River interactions, as shown in Figure 9. The simulations indicated that the outflow process may be altered from the natural discharge into the river (i.e., positive values) to frequent backflow occurrence (i.e., negative values; in red dots), although the magnitude of the backflow volume was relatively small. As mentioned previously, the used control-point method of the PLHP is intended to determine the open or close status, and the simulation results showed a total of 25 days with gates closed, and a total of 21 days of backflows occurred in the study period (31 days) (i.e., ~68% of the time; Figure 9). In addition, the backflow events caused by the PLHP may propagate to the entire outflow channels (e.g., from the outlet to the PLHP; see Figure 9). It follows that the backflow of the Yangtze River blocks the lake outflow process, as discussed by Li et al. [19]. Compared to the role of the backflow, the results presented here demonstrated that the PLHP may play a similar role in influencing the lake outflow dynamics, thus altering the river–lake exchanges.

The outcomes from this study provide an important insight regarding the role of the PLHP on the flow behaviors of Poyang Lake, but the PLHP may significantly reduce the water supply for the main stem of the Yangtze River during this storage period (i.e., September–October). As mentioned previously, the discharge of the Yangtze River is also decreased during this period, mainly due to the impoundment of the TGD [15,16]). Considering these backgrounds, both the PLHP and the TGD are more likely to play a combined impact on the water crisis of the Yangtze River, although the PLHP may alleviate the dry condition of the lake.

### 3.4. Impacts on Water Temperature

**Figure 10:**
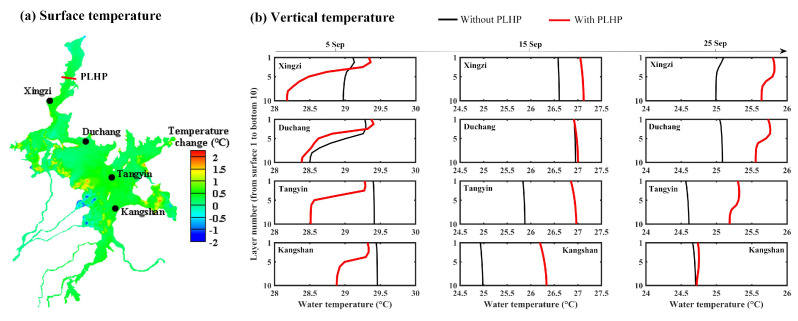
Influence of the PLHP on (**a**) water surface temperature and (**b**) vertical temperature within Poyang Lake. Positive changes in (**a**) indicate that the water temperature in the scenario with PLHP is higher than the natural condition.

Figure 10 shows the spatial influences of the PLHP on water surface temperature and vertical temperature within Poyang Lake. Model simulations indicated that the resulting increase in water surface temperature was observed in the range of ~0.5 to ~1.5 °C for the majority of the lake regions, while a decreasing trend can be found in the catchment river inlets adjacent to the lake shoreline, with a maximum of around −2.0 °C (Figure 10a). Generally, the water surface temperature may exhibit mostly minor temperature changes across the lake. The influence on the simulations resulting from changing the vertical water temperature of the lake is shown in Figure 10b. Although vertical water temperatures at the four gauging stations reacted with relatively complex responses, the PLHP tended to decrease the vertical temperature during the initial period (e.g., 5 September; <1.0 °C) and increase the temperature during other times within the storage period (e.g., 15 September and 25 September; up to 1.0 °C). This is an expected outcome given that the effect of the PLHP on the flow pattern of the lake may exert substantial effects on the water temperature during the initial period, while the meteorological forces may play a critical role in influencing the vertical water temperature due to a gradually increase in the water level of the lake [38].

## 4. Conclusions

The present work aimed to extend previous modeling studies and overcome the limitations of the PLHP conceptualization in the large floodplain system of Poyang Lake. The model simulations provide important information about the effects of the PLHP on the lake hydrodynamics during the study period. Generally, the modeling results showed that the water levels across most of the lake regions presented more significant changes than in the floodplain areas. The magnitude of increased water levels upstream of the PLHP (~1.0 m) was distinctly higher than that downstream (~0.1 m). The PLHP was also likely to decrease the magnitude of the flow velocities in the lake’s flow channels, whereas velocities may experience mostly minor changes throughout the lake’s floodplains. The modifying flow dynamics and transport trajectories resulted in substantial changes in flow direction and pathways in terms of eddy size and number. In addition, the simulations showed that the outflow process caused by the PLHP may be altered from the natural discharge into the Yangtze River to frequent backflow occurrence in the study period. The backflow events may propagate through the entire outflow channels, and hence, significantly reduce the water supply for the main stem of the Yangtze River during the storage period. The PLHP and TGD are more likely to play a combined role in determining the water crisis of the Yangtze River, although the PLHP may alleviate the dry condition of the lake. On average, the lake water temperature may exhibit mostly minor changes (~<1.0 °C) for both the horizontal and vertical directions.

Our results highlight the important role of the PLHP on Poyang Lake during the storage period and the non-negligible effect on water supply for the downstream Yangtze River. The outcomes from this study play a critical role in giving proposals to manage the lake–river interactions, floodplain behaviors, and associated ecological functions. Future research into the effects of the PLHP on Poyang Lake should investigate other regulation periods of the PLHP under different hydrological years (e.g., dry and flood years), given that the current study focuses on only a single storage period. We must acknowledge that the PLHP may generate non-neglectable effects on the lake morphology and the associated hydrodynamic responses during a long operation period. Consequently, more work is needed to combine the current lake hydrodynamic model with sediment transport models so that the extended model includes regions of Poyang Lake and the Yangtze River that are adjacent to the lake outlet, to perform a more detailed analysis of the water and sediment budget under human impacts (e.g., the PLHP and the TGD). Additionally, further research is needed to provide some new insights regarding the dam’s effects on surface and subsurface connectivity across the lake-floodplains.

## Data Availability

The data presented in this study are available on request from the corresponding author.
